# Are Quality-Adjusted Life Years a Good Proxy Measure of Individual Capabilities?

**DOI:** 10.1007/s40273-017-0495-3

**Published:** 2017-02-25

**Authors:** Paul Mark Mitchell, Sridhar Venkatapuram, Jeff Richardson, Angelo Iezzi, Joanna Coast

**Affiliations:** 1 Health Economics at Bristol (HEB), School of Social and Community Medicine, University of Bristol, Bristol, UK; 2 The National Institute for Health Research Collaboration for Leadership in Applied Health Research and Care West (NIHR CLAHRC West), University Hospitals Bristol NHS Foundation Trust, Bristol, UK; 3 UK Renal Registry, Southmead Hospital, Bristol, UK; 4 Department of Global Health and Social Medicine, King’s College London, London, UK; 5 Centre for Health Economics, Monash University, Melbourne, VIC Australia

## Abstract

**Background:**

There is a debate in the health economics literature concerning the most appropriate way of applying Amartya Sen’s capability approach in economic evaluation studies. Some suggest that quality-adjusted life years (QALYs) alone are adequate while others argue that this approach is too narrow and that direct measures of capability wellbeing provide a more extensive application of Sen’s paradigm.

**Objective:**

This paper empirically explores whether QALYs provide a good proxy for individual capabilities.

**Methods:**

Data is taken from a multinational cross-sectional survey of individuals with seven health conditions (asthma, arthritis, cancer, depression, diabetes, hearing loss, heart disease) and a healthy population. Each individual completed the ICECAP-A measure of capability wellbeing for adults and six health utility instruments that are used to generate QALYs, including EQ-5D and SF-6D. Primary analysis examines how well health utility instruments can explain variation in the ICECAP-A using ordinary least squares regression.

**Results:**

The findings show that all seven health conditions have a negative association on overall capability as measured by the ICECAP-A index. Inclusion of health utility instruments into separate regressions improves the predictive power of capability but on average, explains less than half of the variation in capability wellbeing. Individuals with arthritis appear to be less inhibited in terms of capability losses when accounting for health utility, yet those who have depression record significant reductions in capability relative to the healthy population even after accounting for the most commonly used health utility instruments.

**Conclusion:**

The study therefore casts doubt on the ability of QALYs to act as a reliable proxy measure of individuals’ capability.

## Key Points for Decision Makers

**Table:** 

This study adds empirical evidence to the debate in health economics as to whether the quality-adjusted life years (QALY) can provide a comprehensive outcome of societal welfare benefit as well as a measure of life years adjusted for health-related quality of life.
We find that the commonly used measures to generate QALYs, like EQ-5D and SF-6D, perform relatively poorly in explaining individual capability wellbeing, measured using the ICECAP-A capability index.
This study therefore casts doubt over whether QALYs as commonly constructed provide a good proxy of individuals’ broader capabilities, as has been previously argued.

## Background

Nobel-prize-winning economist, Amartya Sen, provided a viable alternative to welfare economic analyses through shifting the evaluative space from a solitary focus on utilities, in terms of desire fulfilment, happiness or life satisfaction, to individual capabilities [1–4]. Capabilities represent the practical opportunities or choices available to realise valuable states of being, also known as ‘functionings’. These include achievements such as good health, adequate nourishment and adequate shelter [5]. Although the theoretical application of the capability approach to health is not new in the economics literature and dates back over a quarter a century [6, 7], there has been a notable increase in interest in more recent times in terms of its normative relevance for health economics [8–12], global health policy [13] and health care ethics [14, 15]. Simultaneously, there have been efforts to measure capability directly, particularly within health economics for the purpose of assessing benefits from interventions, with a number of different measurement instruments being developed in health and social care, public health and mental health [16].

Early views amongst health economists were that the capability approach offered additional insights compared with methods based upon welfare economics [6], which assumes that social welfare is a function of utility, meaning individual preferences. The product combination of health-related utility with life years results in quality-adjusted life years (QALYs) or disability-adjusted life years (DALYs) when disutility is used in conjunction with life years [17]. In the case of QALYs, utility has increasingly been measured using one of a limited number of instruments [18]. These preserve the primary focus upon utility but due to the relaxation of a number of welfare economic principles, health economic evaluations using QALYs derived in this manner have been more commonly described as being theoretically based on ‘extra welfarism’ [19, 20]. This deviation from strict welfare economic theory has sometimes been misunderstood as a direct application of Sen’s capability approach in its entirety, with it being utilised in part to justify the use of outcomes of health-related morbidity and mortality like QALYs [6] and DALYs [21] in health economic analysis. For scholars advocating the use of both the capability approach [22] and welfare economics [23] in health economic evaluations, this claim is disputed.

Sen’s capability approach is notably underspecified in how it should be applied to aid public policy making [24], and this proves to be both a benefit and a disadvantage. It is beneficial in terms of flexibility, allowing a breadth of application across disparate fields such as health, education and technology [25]. However, this under-specification also causes problems when proposing alternatives in which a ‘reference case’ analysis is favoured by decision makers, such as the recommended economic evaluation format taken by the National Institute for Health and Care Excellence (NICE) in the UK [26].

Individual capability should be included in health economic analyses; whether the focus should be only upon people’s *achievements*—their ‘functionings’—or people’s *capability to achieve* is contested. Sen’s example of fasting versus starving serves as a key example for focusing on capability: two people, one of whom is starving and the other of whom is fasting, have comparable functioning in terms of nourishment, but their capabilities to be nourished are notably different. The argument is that focusing on functionings alone would miss important distinctions such as freedoms and choices between individuals [27].

In health economics, whether the focus should be on functioning achievement or the capability to achieve has been widely debated. Cookson [9] and Bleichrodt and Quiggin [11] have argued for the orthodox extra-welfarist approach, relying on the QALY as a best estimate or surrogate measure of a person’s wider capability set (i.e. the vector of functionings that an individual can choose). An alternative argument has been made that the reliance on QALY outcomes focused on health gain is too narrow a focus to capture the full benefits of interventions from health and social care [10], with capabilities measured directly also appearing to be a fuller implementation of the approach [28].

The theoretical dispute concerning the relevance of the capability approach for health economic outcomes is only important if newly developed capability measures give different empirical results which offer additional information when compared with measures of health, such as the EuroQol instrument, EQ-5D [29], and other measures used in the conventional QALY approach; that is, capability is empirically distinct from functioning and the content of capability instruments is not subsumed by the content of instruments used to capture changes in quality of life in QALYs. A hypothesis previously suggested that new measures of capability, specifically ICECAP measures [30, 31], are capturing distinct information from traditional ‘health functioning’ measures, with an emphasis instead on what has been described as ‘psychosocial wellbeing’ [32]. This hypothesis has been backed up in another recent study [33], although both analyses were focused on a single patient population and one health utility instrument commonly used to generate QALYs, the EQ-5D [29]. Therefore, the generalisability of this hypothesis requires further investigation across different health condition populations and health utility instruments that can be used to generate QALYs.

In this paper, we aim to address the following question: whether measures used to produce QALYs are a good proxy for the estimation of capability. This will be investigated empirically, using a cross-sectional dataset across seven different health condition groups and a ‘healthy’ population, collected from four of the G20 countries.

## Methods

### Dataset

This study uses data collected as part of the Multi-Instrument Comparison (MIC) dataset, a large study of health, subjective wellbeing and capability measures collected across different population groups and countries. The data survey was conducted by a global panel company, CINT Pty Ltd, using online panels to recruit relevant individuals. Participants consisted of a healthy population (defined as reporting 70 or higher on a 0–100 visual analogue scale measuring overall health) and seven health condition groups where individuals reported having a primary condition of one of the following: asthma, arthritis, cancer, depression, diabetes mellitus, hearing loss and heart disease, across six countries: Australia, Canada, Germany, Norway, United Kingdom (UK) and the United States (US). Quotas were employed to get a representative sample in terms of age, sex and education in the healthy population, while target quotas of 150 individuals per health condition group per country were employed to reach similar numbers of health condition groups within and across countries [34].

This study uses data from one capability wellbeing measure and six health utility instruments, as well as information about the primary health condition (if any) of the respondents. In this study, the focus is on the seven health condition groups from the four countries with large native speakers of English in the MIC dataset (Australia, Canada, UK, US). The ICECAP-A was not included in the Norway sample and the newly translated German ICECAP-A requires validation before comparisons can be made with the English version. Members of the healthy population from the four countries are also included.

### Measures

#### Capability Wellbeing

Developed for assessing health and social-care interventions, the ICEpop CAPability instrument for Adults (ICECAP-A) is a short, self-complete, five-part measure of capability wellbeing, generated through qualitative interviews with members of the UK population sampled to achieve diversity in terms of socio-economic status, ethnicity and rural/urban classification [31]. The five capabilities captured by ICECAP-A are phrased as “being able to be/have”. They attempt to capture broad concepts related to people’s capability to live a life that they value and they comprise *stability* (‘settled and secure’), *attachment* (‘love, friendship and support’), *autonomy* (‘independent’), *achievement* (‘achieve and progress’) and *enjoyment* (‘enjoyment and pleasure’). The *stability* attribute concerns informants’ desire for continuity in their lives in relation to friends, work and location. The *attachment* attribute emphasises how informants placed emphasis on love, support and social contact. The *autonomy* attribute reflects a desire to be one’s own person and not a liability to others. The *achievement* attribute represents how informants placed value on moving forward in life and attaining their goals. Finally, the *enjoyment* attribute captures everyday enjoyment that people want to be able to have in their lives [31].

The ICECAP-A represents the only attempt as yet to develop a generic capability index that could be used across a broad range of adult patient groups and populations. Conceptually, therefore, it is comparable to generic health utility measures such as the EQ-5D and the SF-6D, which are recommended for use in economic evaluations as they are not focused on specific conditions and therefore have the ability to assist with allocative decisions across a wide range of interventions (within the health sector). One of the distinguishing characteristics of the ICECAP-A measure (and the related ICECAP-O for older people [30]) is that it contains no direct mention of physical health. Although this may be of concern for clinical trials focusing on physical health, it does permit a comparison of capability wellbeing across public bodies such as education, justice, social care and other areas that may influence the demand for health care services.

A number of studies have now been conducted using the ICECAP-A. These include studies of construct validity [35], content validity among members of the public [36], content validity among research professionals [37], and test–retest reliability [38]. Evidence is also beginning to emerge with respect to the responsiveness of the measure in patient groups [39], as well as the impact of different health conditions on capability [40]. Values for the relative importance of capability levels were determined through a best–worst scaling discrete choice experiment (DCE) with members from the general UK population [41]. The index for capability scores is anchored on a ‘no capability–full capability’ 0–1 scale, in which 1 represents ‘full capability’, the highest level of capability on all attributes, and 0 represents ‘no capability’ on all attributes.

#### Health Utility Instruments

Six health utility instruments that can be used to generate QALYs are included in this study. The EuroQol instrument, EQ-5D-5L, consists of five dimensions of health-related quality of life in terms of a person’s mobility, self-care, usual activities, pain/discomfort and anxiety/depression. The original measure consisted of three levels across the five dimensions (EQ-5D-3L) [29]; the measure has recently been updated to include five levels with an aim to improve sensitivity and limit the ceiling effects experienced with the three-level version [42]. The SF-6D is a shortened preference-based version of the Short Form 36-item, ranging from three to six levels, across six dimensions: physical functioning, role limitations, social functioning, pain, mental health and vitality [43]. The Health Utilities Index Mark 3 (HUI3) is a Canadian health utility measure consisting of eight dimensions: vision, hearing, speech, ambulation, dexterity, emotion, cognition and pain. Each dimension has one item per dimension with five or six levels per item [44]. The Assessment of Quality of Life-Eight Dimensions (AQoL-8D) is a newly developed 35-item health utility instrument from Australia, consisting of two super dimensions of physical and mental health or eight dimensions: independent living, pain, senses, mental health, happiness, coping, relationships and self-worth. There is a primary focus on psychological quality of life in the AQoL-8D measure [34]. The 15D health utility instrument was developed in Finland and consists of 15 items: mobility, vision, hearing, breathing, sleeping, eating, speech, elimination, usual activities, mental function, discomfort and symptoms, depression, distress, vitality and usual activity [45]. The Quality of Wellbeing Scale (QWB) consists of a lengthy list of items that capture three aspects of functioning: mobility, social activity and physical activity in combination with questions on symptoms [46].

The methods for eliciting population preferences for health states from the different health utility instruments vary, with a reliance on a visual analogue scale (VAS) for the 15D [45] and QWB [47], standard gamble for the HUI3 [44] and SF-6D [43], a combination of time trade-off (TTO) and DCE for EQ-5D-5L [48], and a VAS/TTO combination for AQoL-8D [49]. All six health-related utility instruments rely on population preferences for eliciting utilities, although the AQoL-8D values are generated from a combination of public and mental health patient preferences [49]. In cases where more than one value set exists for different measures, given that there is currently only an ICECAP-A tariff available for the UK, we use the UK tariffs for the other instruments where available (i.e. EQ-5D-5L and SF-6D) to retain comparability.

### Analysis

The main analysis in this paper aims to examine the relationship between capability wellbeing and health utility and, more specifically, how much of the variation in the ICECAP-A index can be explained by the health utility instruments described above. A number of ordinary least squares (OLS) regressions were undertaken to test this relationship, where we assume a linear relationship between the ICECAP-A index and a number of independent variables. All regressions were tested for OLS assumptions concerning normality, heteroscedasticity, multicollinearity and linearity. Following similar methods to studies estimating subjective well-being from health utility measures [50, 51], two regression model structures are employed:

1 $${\text{ICECAP-A index }} = \, a_{i} \left( {\text{health condition group}} \right) \, + {\text{ socio-demographic controls}}$$

2 $${\text{ICECAP-A index }} = \, a_{i} \left( {\text{health condition group}} \right) \, + \, b_{i} \left( {\text{health utility measure}} \right) \, + {\text{ socio-demographic controls}}$$

The dependent variable in all regressions is the overall value of the ICECAP-A index. Equation (1) describes the direct association of the health conditions on capability, controlling for sex, education, country of residence and age. Reference variables for health condition, sex, education, country of residence and age are the healthy population, being female, highest education being no more than secondary level, residing in the UK and being 18–24 years old. Coefficients reported in these regressions therefore represent the average differences in the capabilities of those with different health conditions relative to the healthy population and the other confounding dummy variables. Based on previous research on the construct validity of the ICECAP-A with the general population [35], we expect that regressions excluding the health utility instruments will show that there is a negative association of health conditions on capability, a positive association of higher education on capability and no association of age or sex on capability. No previous studies are available to suggest the likely impact of residing in different countries on capability measured by ICECAP-A.

Each of the health utility instruments is then added separately into the initial regression to gauge, primarily, the extent to which they capture the health condition association with capability. The results can be interpreted as follows: if an independent variable from Eq. (1) remains statistically significantly different from zero (±) once a health utility instrument is added to Eq. (2), the health utility instrument does not fully capture the health condition association with the overall capability score; if a variable becomes insignificant when a health utility instrument is added, the association of the condition on capability is being captured by the health utility instrument; if a variable changes sign and significance, the health condition has a larger association with health utility compared with overall capability.

Additional statistical analysis on mean health and capability scores, distribution of capability scores, and correlation analysis between health and capability scores were also conducted. All analysis was conducted using STATA.

## Results

**Fig. 1:**
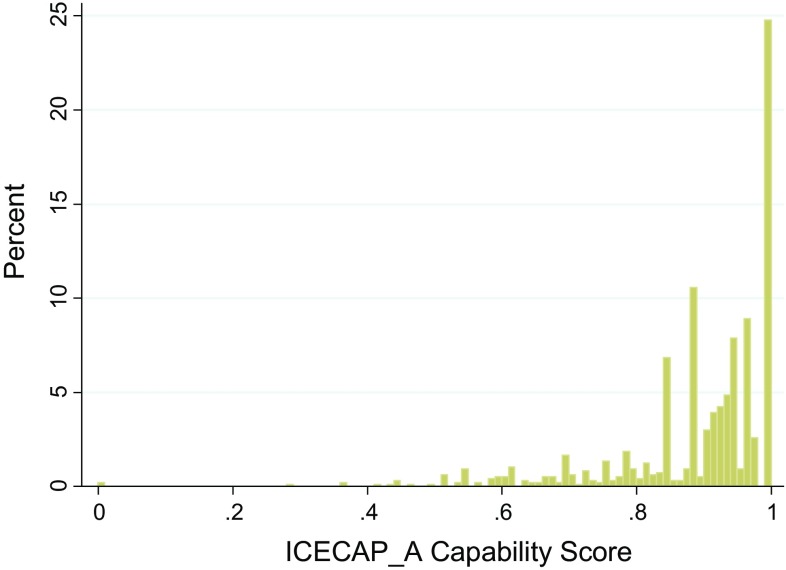
Histogram of ICECAP-A distribution in the healthy population (*n* = 965). *ICECAP-A* ICEpop CAPability instrument for Adults

**Fig. 2:**
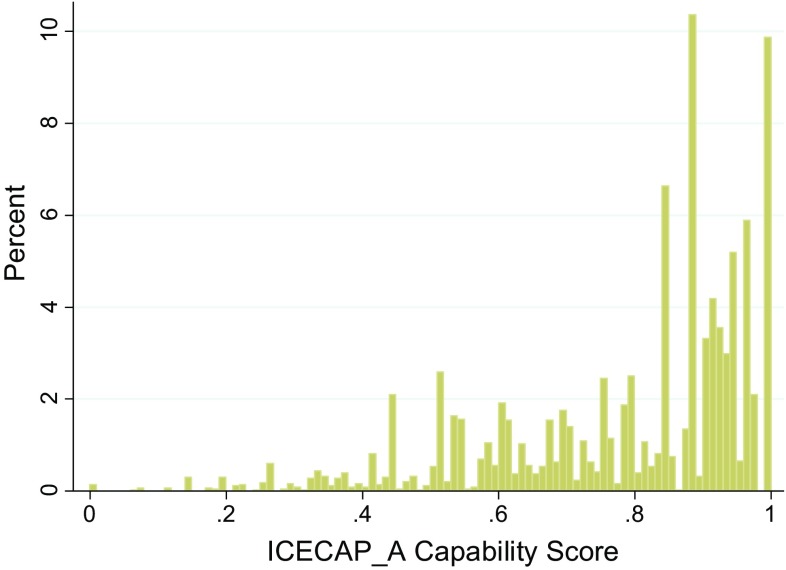
Histogram of ICECAP-A distribution in the health condition population (*n* = 4295). *ICECAP-A* ICEpop CAPability instrument for Adults

In total, 5240 individuals (4295 from the health condition groups and 965 from the healthy population) are included in this study. Individuals excluded for this analysis include people who reported other conditions (*n* = 336). Further information on the inclusion criteria applied to the data prior to this analysis being undertaken can be found elsewhere [51]. Table 1 highlights some of the key socio-demographic information for the individuals included in this study, including sex, highest education attainment, country of residence and age group. Table 2 reports the mean scores across the health and capability measures for the eight population groups. Figures 1 and 2 show the distribution of capability scores for the healthy population and health condition population groups. Table 2 also shows the results of the correlation between ICECAP-A and the six health scores. The ICECAP-A and AQoL-8D correlation of 0.80 was considerably higher than the next best correlations with the HUI3 and 15D of 0.67.

**Table 1:** Socio-demographic breakdown (%) of sample by health condition/healthy population

Health condition	Sex	Highest education			Country of residence				Age group						Total (*n*)
	Male	Secondary level	Further/continuing	Higher level	AUS	CAN	UK	USA	18–24	25–34	35–44	45–54	55–64	65+	
Arthritis	31.3	35.9	38.8	25.3	25.5	21.7	24.8	28.0	1.4	5.8	9.2	22.7	35.6	25.3	640
Asthma	31.1	30.9	32.5	36.6	24.4	23.8	25.9	25.9	13.8	23.5	23.5	16.8	15.0	7.4	579
Cancer	39.5	34.1	33.8	32.1	26.7	23.9	23.7	25.6	1.2	4.0	8.3	18.4	37.6	30.5	577
Depression	32.6	34.8	34.7	30.5	23.7	23.5	25.6	27.2	10.2	25.0	23.0	24.0	13.8	4.1	617
Diabetes	53.7	34.5	38.2	27.3	26.2	22.5	25.1	26.2	1.4	7.0	9.8	23.9	35.3	22.6	641
Hearing loss	52.7	33.6	34.3	32.2	26.7	24.8	21.7	26.9	3.8	8.6	10.3	17.4	27.7	32.2	581
Heart disease	59.5	35.6	38.6	25.8	23.3	24.1	26.1	26.6	3.6	5.0	6.3	17.0	33.8	34.4	640
Healthy	46.8	37.2	32.1	30.7	20.7	27.9	24.2	27.2	12.6	19.6	19.6	18.7	14.5	15.0	965
Total	43.7	34.8	35.2	30.0	24.4	24.3	24.7	26.7	6.4	12.7	14.1	19.8	26.0	21.0	5240

*AUS* Australia, *CAN* Canada, *UK* United Kingdom, *USA* United States of America

**Table 2:** Capability and health scores (standard deviation) for population groups

	ICECAP-A	EQ-5D	SF-6D	AQoL-8D	HUI3	15D	QWB
Healthy	0.893 (0.13)	0.941 (0.08)	0.802 (0.11)	0.828 (0.15)	0.897 (0.13)	0.950 (0.06)	0.764 (0.14)
Asthma	0.810 (0.17)	0.830 (0.18)	0.700 (0.13)	0.672 (0.21)	0.739 (0.25)	0.839 (0.12)	0.627 (0.14)
Arthritis	0.810 (0.17)	0.731 (0.22)	0.664 (0.13)	0.624 (0.22)	0.599 (0.27)	0.808 (0.12)	0.578 (0.13)
Cancer	0.810 (0.18)	0.787 (0.21)	0.685 (0.13)	0.655 (0.22)	0.676 (0.27)	0.816 (0.13)	0.598 (0.14)
Depression	0.637 (0.22)	0.702 (0.22)	0.603 (0.11)	0.452 (0.19)	0.524 (0.31)	0.757 (0.14)	0.538 (0.13)
Diabetes	0.797 (0.19)	0.776 (0.22)	0.680 (0.14)	0.636 (0.23)	0.648 (0.29)	0.818 (0.13)	0.610 (0.15)
Hearing loss	0.855 (0.16)	0.872 (0.14)	0.749 (0.12)	0.719 (0.20)	0.687 (0.23)	0.875 (0.10)	0.639 (0.12)
Heart disease	0.817 (0.18)	0.786 (0.21)	0.690 (0.13)	0.667 (0.23)	0.678 (0.27)	0.819 (0.14)	0.607 (0.15)
Maximum	1.000	1.000	1.000	1.000	1.000	1.000	1.000
Minimum	0.000	−0.276	0.301	0.105	−0.343	0.253	0.151
Correlation with ICECAP-A		0.613	0.631	0.802	0.669	0.667	0.526

ICECAP-A scores on 0–1 (no capability–full capability) scale. Health scores on 0–1 (dead–full health) scale for use in QALYs

*15D* 15-dimension health utility instrument, *AQoL-8D* Assessment of Quality of Life-8 Dimensions, *EQ-5D* EuroQol-5 Dimensions, *HUI3* Health Utilities Index Mark 3, *ICECAP-A* ICEpop CAPability instrument for Adults, *QWB* Quality of Wellbeing scale, *SF-6D* Short Form-6 Dimensions

Table 3 reports the regression analyses showing the extent to which ICECAP-A values are explained by health condition groups and socio-demographic controls alone (regression 1) and with the addition of each of the six health utility instruments individually (regressions 2–7). No OLS assumptions tested were violated. Looking at the prediction of ICECAP-A without health utility instruments included, the association with capability levels for each of the seven conditions can be seen, ranging on average from a 5% reduction in capability for those suffering hearing loss to almost a 25% reduction in capability for individuals reporting a primary condition of depression. All seven conditions have a significant negative association with capability compared with the healthy population sample. With respect to other prior expectations, the hypothesised associations of higher education having a positive association and sex having an insignificant association hold. Being aged between 35 and 54 years has a significant negative association with capability, all else being equal. Being from Australia, Canada or the US compared with the UK and being over 65 years of age all have a positive association with capability. Based on the variables included in column 2, just over 17% of the variation in the ICECAP-A capability score is explained by the health condition and socio-demographic controls alone.

**Table 3:** Regressions explaining ICECAP-A overall score (*n* = 5240)

Variable	1	2	3	4	5	6	7
Health utility							
EQ-5D		0.528*					
SF-6D			0.794*				
AQOL-8D				0.655*			
HUI3					0.437*		
15D						0.929*	
QWB							0.578*
Arthritis	−0.087*	0.017*	0.024*	0.051*	0.036*	0.037*	0.018*
Asthma	−0.080*	−0.020*	−0.002	0.018*	−0.008	0.024*	−0.003
Cancer	−0.092*	−0.017*	0.005	0.032*	−0.001	0.027*	0.004
Depression	−0.247*	−0.122*	−0.095*	−0.010	−0.085*	−0.071*	−0.120*
Diabetes	−0.098*	−0.017*	0.002	0.033*	0.003	0.018*	−0.009
Hearing loss	−0.050*	−0.015*	−0.002	0.035*	0.040*	0.019*	0.026*
Heart disease	−0.087*	−0.009	0.009	0.032*	0.004	0.032*	0.008
Male	0.000	−0.004	−0.012*	−0.015*	−0.001	−0.009*	−0.016*
University	0.032*	0.012*	0.015*	−0.008*	−0.003	0.003	0.021*
Dip/cert/trade	0.017*	0.008	0.012*	−0.003	0.005	0.005	0.018*
Australia	0.033*	0.012*	0.023*	0.008	0.016*	0.014*	0.026*
USA	0.037*	0.024*	0.029*	0.011*	0.024*	0.026*	0.032*
Canada	0.040*	0.021*	0.022*	0.007	0.019*	0.022*	0.030*
Age 25–34 year	−0.008	−0.001	−0.011	−0.002	−0.002	0.004	−0.013
Age 35–44 year	−0.032*	−0.007	−0.029*	−0.013	−0.007	−0.002	−0.028*
Age 45–54 year	−0.040*	−0.001	−0.035*	−0.015*	−0.001	0.003	−0.032*
Age 55–64 year	−0.011	0.023*	−0.018*	−0.013	0.021*	0.026*	−0.009
Age 65+ year	0.044*	0.057*	0.017	−0.005	0.059*	0.060*	0.030*
Constant	0.860*	0.367*	0.249*	0.362*	0.475*	−0.019	0.435*
Adjusted *R* ^2^	0.173	0.442	0.443	0.655	0.510	0.498	0.351

*15D* 15-Dimension health utility instrument, *AQoL-8D* Assessment of Quality of Life-8 Dimensions, *EQ-5D* EuroQol-5 Dimensions, *HUI3* Health Utilities Index Mark 3, *ICECAP-A* ICEpop CAPability instrument for Adults, *QWB* Quality of Wellbeing scale, *SF-6D* Short Form-6 Dimensions

* Statistically significant where *p* ≤ 0.05

In regressions 2–7 in Table 3, the six health utility instruments are added to the regression separately. For the arthritis population, all six health utility regressions report a positive significant coefficient, suggesting the condition has a greater association with the six health utility measures than capability as captured by ICECAP-A. The same trend is observed for hearing loss on four of the regressions including health utility measures (not for regressions including EQ-5D or SF-6D). For people with depression, five of the six measures produced negative significant coefficients, underestimating the impact of depression on capability captured by the ICECAP-A. The addition of AQoL-8D to the regression (Table 3, regression 4) turns six health condition variables (except depression) to positive significant variables, suggesting a larger association with the AQoL-8D than capability. A similar trend is recorded on the 15D for the same six health condition groups.

The addition of EQ-5D and SF-6D to the regression adds less explanatory power to the ICECAP-A scores compared with the AQoL-8D, HUI3 and 15D instruments, respectively. The AQoL-8D regression explains variation in ICECAP-A values best, with an adjusted *R* ^2^ of 0.655, compared with an average adjusted *R* ^2^ of 0.48 across the six regressions including the health utility instruments.

## Discussion

In this study, the debate surrounding whether the QALY provides a good proxy for measuring individual capability is empirically tested, using six health-utility instruments and a measure of perceived capability wellbeing across four countries, seven health conditions and a healthy population sample. The main findings of this study show that all health conditions studied here have negative associations with capability wellbeing compared with the healthy population, ranging from a 5% decrement for those with hearing loss to a 25% reduction for those with depression. On average, the six regressions including the most common health utility instruments applied in economic evaluation do not explain the majority of variation associated with capability well-being as measured by the ICECAP-A. The EQ-5D and SF-6D, the most frequently used health utility instruments [52], perform poorly in explaining variation in capability wellbeing relative to the regressions including the newly developed AQoL-8D and to a considerably lesser extent, the HUI3 and 15D.

This study examined health utility and capability wellbeing across a wide variety of health conditions and four nations with differing healthcare systems, so the results benefit from this level of comprehensiveness. The large number of health utility instruments is also an important strength, allowing conclusions to be drawn for more than one interpretation of health; it was not possible to achieve similar comprehensiveness in relation to capability instruments as there is only one such instrument available for the adult population.

There are a number of limitations associated with this dataset, namely the cross-sectional nature of data currently available and that population groups are split by broad health condition categories. Therefore, we are unable to assess important issues related to health status and capability wellbeing captured on the ICECAP-A with regards to longitudinal changes of capability over time and whether improvements in health conditions similarly or differently effect health utility or capability.

Values for estimating overall scores for the six health utility instruments and one capability wellbeing instrument were derived from those currently available. However, the conceptual differences embedded in the descriptive systems of measures are likely to be of greater importance than differences in valuation across countries. Separate analyses of the six health utility instruments confirms that differences are primarily a result of the descriptive systems and not the weights applied [53]. It should be noted that an implicit assumption in the work is that ICECAP-A provides a strong measure of capability: it is clearly difficult to test this assumption given that other generic measures were not available here. Whether or not capabilities can be self-reported remains a lively debate in the capability community, as the capability approach was developed in part to reduce subjective adaptation in utility measurement [20].

This study has questioned whether measures of health utility are able to explain capability wellbeing adequately and whether QALYs created from existing health utility instruments provide a good proxy measure of capability. Although this study generates some evidence that health utility measures are able to explain the health condition component of capability wellbeing, particularly for physical health conditions, the regressions including a measure of health utility failed to explain, on average, half of the variation in capability wellbeing scores across the broad sample surveyed here (i.e. mean average adjusted *R* ^2^ of 0.48 across six regressions ranging from 0.35 to 0.66; see Table 3).

The impact on capability for individuals with a primary health condition of depression is underestimated by the majority of commonly used health utility instruments. The only measure that captures the capability reduction from depression is the newly developed AQoL-8D, which has primarily aimed to redress the perceived imbalance in existing measures against psychosocial health [34]. The performance of the more commonly used health utility instruments in this analysis adds support to a belief that mental health is unfairly treated using the QALY [54, 55], and that has led to some researchers developing a capability measure for mental health patients [56]. The findings here will similarly support the consensus of other researchers who have made similar criticisms of the use of the QALY in non-healthcare settings such as social care [57], public health [58], end-of-life care [59] and other complex interventions [60].

This study focused on one main difference between those advocating a more extensive use of the capability approach and those committed to the extra-welfarism approach currently practiced in health economics. Differences exist, not only in measurement, but also in decision rules and valuation where the extra-welfarism commonly applied remains inherently welfarist in practice [23, 61]. Progress has been made in developing a capability approach alternative to standard practice in terms of measures of capability [16, 62], decision rules by moving towards a sufficient capability objective [63, 64], and valuation with best–worst scaling DCE offering a mechanism for estimating the relative importance of different capability states [30]. Further research is still required, particularly on how a unit of capability gain, however defined, is monetarily valued before a fully workable alternative to the conventional QALY approach can be provided to decision makers. Further research is also required to understand how measures of perceived capability like ICECAP-A are susceptible to adaptation over time.

## Conclusion

This study has contributed to the growing literature which seeks to demonstrate the role and value of capabilities in the analysis of health and related sectors where presently QALYs are the only economic outcome deemed to be relevant. Specifically it tested, empirically, whether or not health utilities used to create QALYs could satisfactorily measure capabilities across seven common health conditions. The health utility instruments included in this study were found to have significant but variable explanatory power depending on the measure used. Nevertheless, none of the instruments fully predicted or explained levels of capability wellbeing across a number of health conditions. Some of the lowest explanatory powers of capability in regression analysis undertaken here were those that included the most commonly used health utility instruments, the EQ-5D and SF-6D. This observation provides support for the addition of information concerning capabilities in evaluation studies when these health utility instruments are used.
