# Correction to: Are Quality-Adjusted Life Years a Good Proxy Measure of Individual Capabilities?

**DOI:** 10.1007/s40273-018-00763-4

**Published:** 2019-03-09

**Authors:** Paul Mark Mitchell, Sridhar Venkatapuram, Jeff Richardson, Angelo Iezzi, Joanna Coast

**Affiliations:** 1 Health Economics at Bristol (HEB), School of Social and Community Medicine, University of Bristol, Bristol, UK; 2 The National Institute for Health Research Collaboration for Leadership in Applied Health Research and Care West (NIHR CLAHRC West), University Hospitals Bristol NHS Foundation Trust, Bristol, UK; 3 UK Renal Registry, Southmead Hospital, Bristol, UK; 4 Department of Global Health and Social Medicine, King’s College London, London, UK; 5 Centre for Health Economics, Monash University, Melbourne, VIC Australia

## **Correction to:** PharmacoEconomics (2017) 35:637–346 10.1007/s40273-017-0495-3

The Open Access license, which previously read:

**Open Access** This article is distributed under the terms of the Creative Commons Attribution-NonCommercial 4.0 International License (http://creativecommons.org/licenses/by-nc/4.0/), which permits any noncommercial use, distribution,and reproduction in any medium, provided you give appropriate credit to the original author(s) and the source, provide a link to the Creative Commons license, and indicate if changes were made.

Should read:

**Open Access** This article is distributed under the terms of the Creative Commons Attribution 4.0 International License (http://creativecommons.org/licenses/by/4.0/), which permits unrestricted use, distribution, and reproduction in any medium, provided you give appropriate credit to the original author(s) and the source, provide a link to the Creative Commons license, and indicate if changes were made.

The original article has been corrected.

